# The role of *Helianthus tuberosus* powder in healing of full-thickness wounds in mice

**DOI:** 10.14202/vetworld.2021.1290-1298

**Published:** 2021-05-24

**Authors:** Ali Ghazi Atiyah, Nadia Hameed Rija AL-Falahi

**Affiliations:** 1 Department of Surgery and Obstetrics, College of Veterinary Medicine, University of Tikrit, Iraq; 2 Department of Surgery and Obstetrics, College of Veterinary Medicine, University of Baghdad. Iraq

**Keywords:** antimicrobial activity, fatty acids, Jerusalem artichoke, medicinal plants, wound healing

## Abstract

**Background and Aim:**

Recently, many medicinal plants have received considerable attention in the medical field because of their role in the wound healing potential. This study aimed to determine the effectiveness of *H. tuberosus* powder on the healing pathway of full-thickness cutaneous wounds in a mouse model.

**Materials and Methods:**

*H. tuberosus* powder was prepared by a freeze-drying process using a lyophilizer and its active ingredients were evaluated by high-performance liquid chromatography (HPLC), while its antibacterial properties were evaluated by agar well diffusion assay. The percentage wound contraction was also assessed. Thirty mice were used, which were divided equally into two groups, a control group and a treated group. A full-thickness wound, 1 cm×1 cm in size, was established on the dorsal aspect of the thoracolumbar region, into which *H. tuberosus* powder was topically applied in the treated group. In contrast, the control group was left without any treatment. The animals were euthanized on days 7, 14, and 21 after wounding for histopathological study.

**Results:**

The agar well diffusion method indicated the antibacterial activity of *H. tuberosus*, while the HPLC results indicated that the active ingredients of *H. tuberosus* powder mainly consisted of three major kinds of fatty acid. In addition, the macroscopic results of wound contraction rate and the histopathological outcomes of the healing process were significantly (p≤0.05) enhanced in the treated group compared with those in the control group.

**Conclusion:**

*H. tuberosus* powder acts as an antibacterial agent with the ability to enhance the wound healing process.

## Introduction

The healing of cutaneous wounds is a complex biological process by which skin repairs itself after injury. This process consists of three highly integrated steps: Inflammation, tissue proliferation, and tissue remodeling. It involves the activation of inflammatory cells, endothelial cells, keratinocytes, and fibroblasts, along with the synthesis and liberation of active mediators (cytokines and growth factors) to regulate tissue repair and regeneration [1]. These steps and their physiological constituents should occur in the appropriate sequence and at certain times to achieve optimal function and the tensile strength of wounded area of the original skin [2]. Different approaches are used to promote skin wound healing, such as the administration of local or systemic antibiotics or the use of antiseptic solutions [3]. The increasing pathogenic resistance against currently used synthetic medicinal agents, such as antibiotics, antifungal, and antiviral products, has prompted interest among many researchers in the identification of novel antibacterial compounds originating from plants, plant-based phytochemicals as possible alternative to antibiotics which are still used in modern and traditional systems of medicine for the treatment of the disease [4]. Recently, plants or chemical compounds derived from plants have been used as pharmaceutical agents for treating cutaneous wounds. Medicinal plants play roles in wound healing through many different mechanisms, such as decreasing the bacterial count, enhancing angiogenesis, accelerating the deposition of collagen, and increasing fibroblast proliferation [5,6].

The biological properties of medicinal plants influence key events in mammalian wound healing, playing a key role in regulation of the wound healing pathway. They are thus considered as a source of many macromolecules such as carbohydrates, fatty acids, and proteins that can be used as biomaterials for developing new candidate drugs to promote wound healing [7,8]. About 500,000 plant species exist globally, among which only about 1% have been studied by researchers [9].

*Helianthus tuberosus*, the Jerusalem artichoke, is a sunflower that emerged in eastern North America and is now widely distributed in the Middle East, especially Iraq. It is 2–3 mm in length with superficial leaves and plump tubers [10,11]. It has been established that this species exhibits several medical activities, such as purgative, diuretic, and bowel tonic effects, and has been used as a folk medicine for managing bone fractures and cutaneous wounds, and even for relieving pain [12]. Various studies have also found that *H. tuberosus* compounds have antioxidant, anti-inflammatory, antimicrobial, antifungal, anticancer, antipyretic, and analgesic effects [13-15]. Jerusalem artichoke is rich in fatty acids such as palmitic acid, which resemble those present in the sebaceous glands in the skin [16]. These fatty acids in this plant have been reported to possess potent antimicrobial–disinfectant properties in the skin [17]. Against this background,

This study aimed to determine the effectiveness of *H. tuberosus* powder on the healing pathway of full-thickness cutaneous wounds in a mouse model.

## Materials and Methods

### Ethical approval

The animal utilization protocol of the experiment was approved by the Animal Care and Use Committee, College of Veterinary Medicine, University of Baghdad, Iraq, with approval number 1929 dated 17-12-2020.

### Study period and location

The study was conducted from January to December 2020. The samples were processed at Laboratories of the Animal House Center in the College of Veterinary Medicine, Baghdad University.

### Preparation of *H. tuberosus* powder

*H. tuberosus* was purchased from a local market in Baghdad, Iraq, during the January 2020. The plant was identified and authenticated by Professor Dr. Rana H. Aloush AL- Sammarai, a botanist in the Department of Biology, College of Sciences, University of Tikrit. Iraq. Preparation of *H. tuberosus* powder was performed in accordance with a modified version of a previously reported method [18]. The plants were washed and cleaned under tap water to eliminate soil or any debris and then longitudinally sliced to obtain approximately 2-mm-thick pieces using a stainless steel knife. After that, the samples were transported to the laboratory for a freeze-drying process using a lyophilizer ( LG-101;Nabel, Hangzhou, China) to remove moisture from the plants and ensure their dryness. The dried slices of *H. tuberosus* were ground into a fine powder 10 mm in size with a laboratory mortar grinder (Retech-Rm200, India). The final product was a pale-brown fine powder with an aromatic smell, and the product was kept at room temperature in a sterile plastic package until used [18].

### Antimicrobial evaluation

The antimicrobial activity of *H. tuberosus* was evaluated by agar well diffusion method, as described previously [19]. The bacterial isolates from the contaminated wounds were *Staphylococcus aureus*, *Pseudomonas aeruginosa*, *Klebsiella pneumonia*, and *Proteus mirabilis*. All of these microorganisms were incubated in Muller–Hinton broth overnight at 37°C (the turbidity adjusted to 0.5 McFarland standards) giving 10^6^ colony-forming units (CFU) mL-¹ in sterile phosphate-buffered saline. Then, 25 mL of nutrient agar at 50°C was added in each Petri dish and 100 μL of the diluted (10^6^ CFU) bacterial broth was added into the Petri dishes using a sanitized spreader. 10 min later, a well 5 mm in diameter was made in the center of the plate with a sterilized cork borer. Next, 40 μL of *H. tuberosus* solution at four desired concentrations (100%, 50%, 25%, and 10%) was loaded in the hole and the zones of inhibition were measured in millimeters using a ruler. The inoculated plates were then incubated at 37°C overnight [20]. All of the above steps were carried out in a laminar flow hood.

### High-performance liquid chromatography (HPLC)

To determine the main components of *H. tuberosus* powder samples, liquid chromatography using a Shimadzu 10 AV-LC equipped with a binary distribution pump model (LC-10A Shimadzu) was used. The eluted peaks were monitored by a Shimadzu SPD 10Avp detector and the data were reported on Shimpack C-R8A (Shimadzu, Koyota, Japan).

### Animals

Thirty young adults (7-8 weeks old) Kunming mice weighing 20-25 g were purchased from the animal house at the College of Veterinary Medicine, Baghdad University. The environment in the cages was kept at temperature of 25 ± 2°C, humidity 55-60%, with a 12:12 h light/dark cycle. The animals were fed standard pellets and tap water. The animals were randomly allocated into two main groups (15 animals per group).

### Full-thickness wound creation

The mice underwent general anesthesia using a combination of 90 mg/kg ketamine (Alfasan, Holland) and 10 mg/kg xylazine (Rampon-Virbac Laboratories, France) intraperitoneally. The dorsal aspect of the thoracolumbar region was surgically prepared and a full-thickness wound 1 cm×1 cm in size was created using a surgical scalpel (blade No.11) on all of the animals. All wounds were left undressed overnight and open to the environment to encourage microbial colonization and proliferation [21]. A skin swab was taken to determine the types of bacteria contaminating the wound before treatment. The animals in the control group were left untreated, while in the treated group the animals were topically administered 50 mg of *H. tuberosus* powder twice daily during the first 5 days after injury. The changes in wound area by contraction were measured on days 1, 3, 7, 10, 14, and 21 after wounding as percentages using a caliper and calculated using the following equation [22].


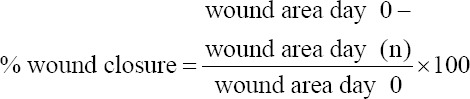


### Macroscopic evaluation

During this study, the wound area in all animals was photographed on days 1, 3, 7, 10, 14, and 21 after wounding to determine any signs of inflammation, erythema, edema, necrosis, and hemorrhage, and the times of wound contraction and closure were also recorded.

### Histopathological evaluation of excised tissue

The wound tissue samples were obtained on days 7, 14, and 21 after injury. Five samples from the animals were collected from each group for histological analysis. The animals were euthanized with a lethal dose of ketamine and xylazine administered intramuscularly. Full-thickness samples were excised using sharp and blunt dissection and then fixed in 10% buffered formalin, dehydrated by alcohol, cleared in xylene, and embedded in paraffin wax. Serial sections of 5 mm in thickness were made using a microtome (Leica SP 1600; Leica Microsystems, Germany) and finally stained with hematoxylin and eosin. The stained sections were observed by a pathologist under a light microscope (Olympus, A × 80T, Japan). In addition, the wound healing process was evaluated semi-qualitatively in accordance with a previously reported method [23] using the histopathology grading scale shown in Table-1.

**Table-1:** Wound histopathology grading scale.

S. No.	Finding	Score	Description
1	Epidermal hyperplasia	0	None
		1	Minimal
		2	Mild
		3	Moderate
		4	Marked
2	Re-epithelization	0	None
		1	Minimal: ≤1/3 of wound surface covered by epithelium
		2	Partial:>1/3 but ≤2/3 of wound surface covered by epithelium
		3	Extensive >2/3 of wound surface mostly covered by epithelium
		4	Complete: All wound surface covered by epithelium
3	Granulation tissue	0	Wound unhealed, devoid of granulation tissue
		1	Wound filled with only immature collagen
		2	Wound filled by immature and mature collagen
		3	Wound mostly filled by mature collagen with some immature
		4	Wound completely filled by mature collagen
4	Inflammatory cell response	0	None
		1	Minimal
		2	Mild
		3	Moderate
		4	Marked
5	Hemorrhage	0	None
		1	Minimal
		2	Mild
		3	Moderate
		4	Marked

### Statistical analysis

The data were collected by calculating the percentage contraction of the wound area in millimeters. Histopathological analysis was also performed in both groups. The presented numerical data are expressed as mean±standard deviation (SD), and the statistical significance of differences between the two groups was analyzed by *t*-test using SAS version 9.4 software (SAS Inst. Inc., Cary, NC, USA.). The differences between groups were considered significant at *P* < 0.05.

## Results

### Antimicrobial evaluation

**Figure-1:**
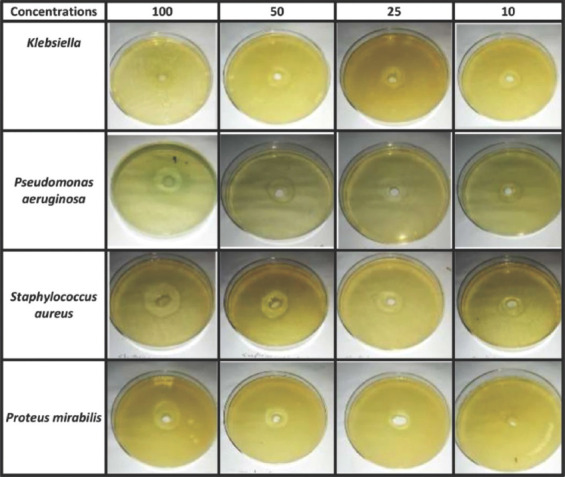
The zones of inhibition diameters in (mm) using agar diffusion methods at different concentrations for determination antibacterial activity of *Helianthus tuberosus* powder against a certain type of bacteria.

The agar well diffusion method revealed elliptical zones around the wells, indicating the antimicrobial activity of *H. tuberosus* powder against pathogenic bacteria that were previously isolated from the contaminated wounds. Photographs of the zones of inhibition of *H. tuberosus* powder against all of the four bacteria are shown in Figure-1. The sizes of the zones on the respective plates as measured using a ruler are shown in Table-2.

**Table-2:** Antibacterial inhibition zone (mm) of *Helianthus tuberosus* powder in the nutrient agar at different concentrations (mg/mL).

S. No.	Bacterial culture	*H. tuberosus* concentration			

		100%	50%	25%	10%
1	*Staphylococcus aureus*	26	24	23	22
2	*Pseudomonas aeruginosa*	25	22	21	20
3	*Klebsiella pneumonia*	25	25	23	20
4	*Proteus mirabilis*	25	22	20	Zero

### HPLC

The HPLC results indicated that the primary components of *H. tuberosus* were fatty acids, mainly consisting of palmitic acid, stearic acid, and myristic acid, at concentrations of 26.15%, 26.08%, and 25.21%, respectively (Table-3).

**Table-3:** HPLC analysis of *Helianthus tuberosus* powder sample extracted by HPLC analysis.

S. No.	Subjects	Reaction time minute	% of fatty acids
1	Palmitic acid C16:1	2.29	26.15
2	Stearic acid C18:0	3.57	26.08
3	Myristic acid C14:0	4.65	25.21

HPLC=High-performance liquid chromatography

### Macroscopic evaluation

**Figure-2:**
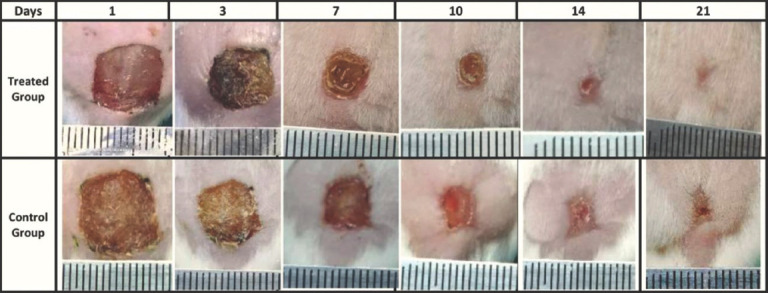
The macroscopic image of the wound contraction in the treated and control group at different intervals.

Signs of inflammation, erythema, and edema were seen in the treated group during the first 3 days after injury. In the control group, these signs were maintained up to the first 10 days after injury. Signs of necrosis and hemorrhage were not seen in either group during the present study. In addition, more rapid contraction of the wound took place in the treated group than in the control group, revealing complete closure of the wounds on day 21 (Figure-2). In terms of the mean wound contraction, there was significant enhancement of contraction (*P* < 0.05) in the treated group compared with that in the control group starting from day 7 (84.43%) to day 21 (10.85%; Table-4).

**Table-4:** Mean values of wound contraction at different intervals time in treated and control wounds.

Values	7 day		14 day		21 day	
		
	Control	Treated	Control	Treated	Control	Treated
Mean	87.77%	84.43%	55.42%	45.44%	27.81%	10.85%
Standard deviation	8.43%	11.10%	17.06%	16.34%	15.56%	12.56%
t-value	2.33*		4.33*		8.68*	

* (p≤0.05)

### Histopathological evaluation

**Figure-3:**
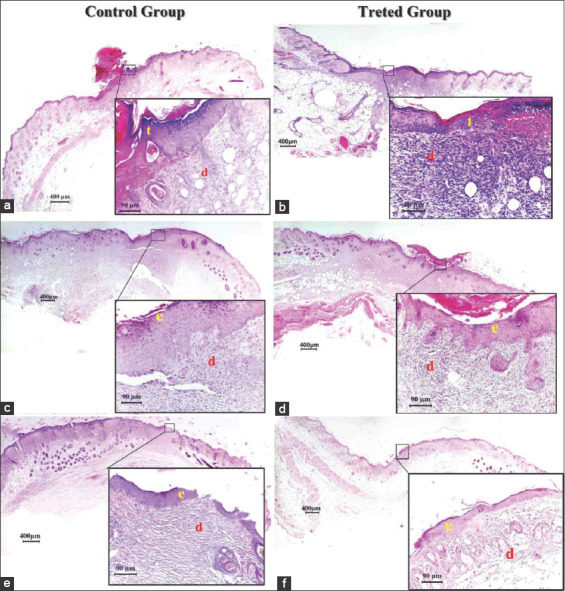
(A) Control group 7 days, thick serocellular crust with small epidermal tongue grow toward the center of wound(t), inflammatory cells(d). (B) Treated group 7 days, thin layer of fibrin covering the wound area with well-developed epithelial tongue to word the center of wound area (t) infiltration of inflammatory cells. (C) Control group 14 days, keratinocytes (e) formation of granulation tissue in the dermal layer (d). (D) Treated group 14 days, thick epidermal layer (e), presence of less dense collagen fibers deposition in the dermal layer (d). (E) control 21 days, delicate epidermal layer (e) short dense irregular collagen fibers in the dermal layer (d). (F) Treated group 21 days, well-organized epidermal layer (e) presence of mature collagen fibers in dermal layer (d). Hematoxylin and eosin staining and the scale bar represents 90 and 400 µm.

**Figure-4:**
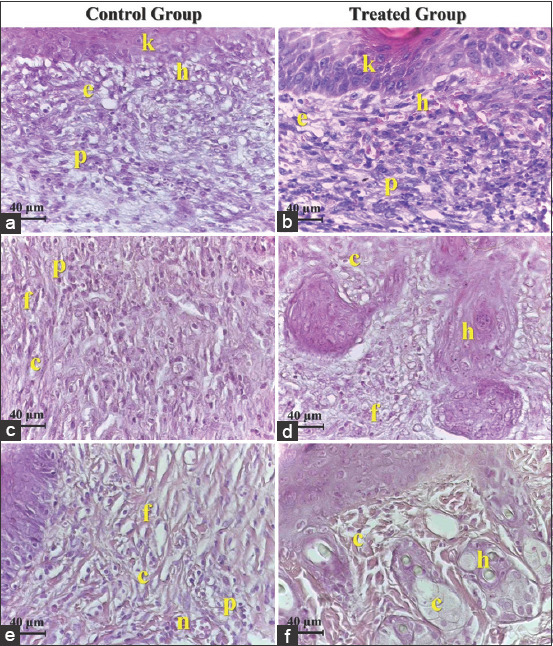
(A) Control group 7 days, large numbers of polymorphonuclear cells (p), hemorrhage (h) with edema (e) keratinocytes (k). (B) Treated group 7 days, large numbers of polymorphonuclear (p), hemorrhage (h) edema (e) keratinocytes under mitotic division (k). (C) Control group 14 days, polymorphonuclear (p), dense irregular orientation of collage fibers (c) fibroblasts (f). (D) Treated group 14 days, less dense collagen fibers (c) fibroblasts (f) newly formed hair follicles (h). (E) Control group 21 days, inflammatory cells (p) short dense collagen fibers (c) fibroblasts (f) blood vessels (n). (F). Treated group 21 days, mature collagen fibers (c) newly formed hair follicles (h) sebaceous gland (s). Hematoxylin and eosin staining and the scale bar represents 40 µm.

Histopathological observation in the control group on day 7 after injury revealed a thick fibrin clot with serocellular crust covering the injured area, with a less developed epidermal tongue at the wound edge (Figure-3A). Other sections showed acute inflammatory reactions through the infiltration of multinucleated cells with hemorrhage, and edema with marked granulation tissue was also seen (Figure-4A). On day 14 after injury, complete re-epithelialization by a thin layer of epithelium had occurred, with the presence of small numbers of active keratinocytes in the epidermal layer (Figure-3C). Other sections indicated new deposition of a small number of irregularly orientated collagen fibers with the infiltration of large numbers of inflammatory cells between these fibers (Figure-4C). On day 21 after injury, the epidermal epithelium was very loose with less stratified squamous epithelium (Figure-3E). Other sections showed the presence of short irregular dense collagen fibers with granulation tissue interposed between fibers in the dermal layer (Figure-4E).

In the treated group, a section on day 7 after injury showed a thin layer of fibrin covering the wound area with the marked proliferation of well-developed epithelial tongue from the wound edge toward the center of the wound (Figure-3B). Other sections showed epithelial and keratinocyte cells that were proliferating and migrating, orientated toward the center of the wound defect with infiltration of the dermal layer by polymorphonuclear cells (Figure-4B). On day 14 after injury, there was complete re-epithelialization by a thick layer of epidermis seen in the wound area, with mild infiltration of polynuclear cells along with the deposition of a large number of collagen fibers with a moderate number of fibroblasts interposed between these fibers in the dermal layer (Figure-3D). Another section revealed newly formed hair follicles with associated sebaceous glands (Figure-4D). On day 14 after injury, the epidermis was well organized by many layers of stratified squamous epithelium and the surface of the stratum corneum featured numerous keratinocytes (Figure-3F). Other sections exhibited mature collagen fibers surrounded by newly formed hair follicles and well-developed sebaceous glands (Figure-4F). The histopathological scores for the two groups indicated a significant increase (p≤0.05) in epidermal hyperplasia in the treated group of Grade 4 on day 7 after injury, which decreased on day 21 to Grade 1. In addition, there were significant differences in hemorrhage on day 7 and day 21. There was a significant increase (p≤0.05) in the full-thickness re-epithelialization in the treated group on days 7, 14, and 21 after injury as compared with the control group. The inflammatory cell response and amount of granulation tissue also showed significant differences between the two groups on days 14 and 21 after injury (Table-5).

**Table-5:** The mean values of histopathological scores for two groups.

S. No.	Finding	7 days			14 days			21 days		
		
		Control	Treated	Value	Control	Treated	Value	Control	Treated	Value
1	Epidermal hyperplasia	0	1	0.52*	2	3	0.52*	2	3	0.50*
2	Re-epithelialization	1	2	0.52*	2	3	0.52*	3	4	0.52*
3	Granulation tissue	1	2	0.52*	1	3	0.52*	2	4	0.52*
4	Inflammatory cell response	4	4	0.00 NS	3	1	0.52*	2	0	0.52*
5	Hemorrhage	2	3	0.52*	1	1	0.00 NS	1	1	0.00 NS

* (p≤0.05), NS=Non-significant

## Discussion

Medicinal plants have been extensively used as alternative wound healing therapies. Many compounds derived from medicinal plants can promote the healing of cutaneous wounds. Therefore, many of these compounds are of interest to researchers in the medical field [24]. The presence of certain phytochemical compounds may be responsible for the antibacterial properties of the Jerusalem artichoke *H. tuberosus*, especially phenols, phenolic acids, flavonoids, isoflavones, and fatty acids. Fatty acids were focused on in the present study because of their physiological role in the healing process [25-27]. The fatty acid compounds identified by HPLC in the present study showed antibacterial activity against Gram-negative bacteria such as *P. aeruginosa*, *K. pneumonia*, and *P. mirabilis*, and against Gram-positive bacteria such as *S. aureus*, when placed on nutrient agar. These results are similar to those in many studies on extracts of fatty acids from plants [28-30]. Thus, the antimicrobial activity of these fatty acids can play significant roles in wound healing, through eliminating infectious agents and initiating normal wound tissue repair.

In the present study, HPLC indicated the presence of three main fatty acids in *H. tuberosus* powder, namely, palmitic, stearic, and myristic acids. A previous study also showed that these fatty acids have the ability to modify the skin structure through maturation and differentiation of the stratum corneum [31]. This was also supported in the current study as the stratum corneum developed over time in the treated group compared with that in the control group. The exact mechanisms behind the antibacterial effects of these fatty acids depend on the number of carbons in the backbone; fatty acids with a backbone longer than 12 carbons have a greater effect against microorganisms than shorter ones [32]. Therefore, the effect of the length of the molecule suggests that the surface-active action of the acid might participate in the dissociation of autolysins from the bacterial cell membrane through inhibiting the activity of cell membrane-associated enzymes, which impairs bacterial growth [33]. In this context, the results of the antimicrobial activity of plant powder in the agar well diffusion method are related to the direct effects of palmitic acid (C16) and myristic acid (C14), as reported previously [34].

Moreover, the histopathological outcomes of the present study revealed that the effect of *H. tuberosus* powder on the treated animals improved the proliferation of fibroblast cells. As a consequence, it increased the production of collagen in the defect area along with developing the tongue of the epithelium containing active epithelial basal cells, resulting in complete full-thickness re-epithelialization at the wound edge without excessive scarring after a skin injury. Similar results have been described previously [35]. In addition, the histopathological findings also revealed complete regeneration of the epidermis, highly regular collagen deposition, angiogenesis, and epithelialization with active keratinocytes in the treated group during the present study, while the control group exhibited delayed healing, indicated by fragile development of the epidermal layers with short, dense irregular deposition of collagen bundles along with the infiltration of large numbers of polymorphonuclear cells in the dermis layer. All of these outcomes may have been due to the potent anti-inflammatory activity of fatty acids by regulating the tissue regeneration process directly in the initial activation of cytokines and growth factors during wound healing [17].

Alexandru *et al*. [36] mentioned that the exogenous antioxidants from plants can reduce oxidative harm through potent free-radical scavengers during wound healing. It has been reported that fatty acids are an important source of natural antioxidants that can play a crucial role in the wound healing process [37]. Other studies have shown that a low concentration of free oxygen radicals in the wound area can increase angiogenesis by stimulating the expression of vascular endothelial growth factor in keratinocytes and macrophages, as well as activate the synthesis of collagen fibers [38,39]. The present histopathological results showed that the topical administration of *H. tuberosus* powder caused a significant increase in the numbers of fibroblasts and collagen fibers along with a considerable decrease in the number of inflammatory cells and the amount of granulation tissue formation in the last period of the present study in the treated group, compared with those in control. These findings match previous results [40], indicating that medicinal plants have mitogenic activity on cellular motility and proliferation. In agreement with this, we reported that the histopathological findings revealed significant epidermal hyperplasia with active epithelial and granular cells of the epidermis under mitotic division in the treated group.

The histopathological scores in the current study revealed significant differences (p≤0.05) in epidermal hyperplasia and re-epithelialization between the two groups. In the treated group, the keratinocytes migrating and proliferating within the wound area started to undergo dense hyper-proliferation in the epithelium directed toward the center of the wound area, while in the control group the re-epithelialization was still fragile and required more time to restore the normal integrity of the epidermal tissue in the wound area. These results match other research, which indicated that the re-epithelialization process is ensured by local keratinocytes at the wound edge and by epithelial stem cells derived from hair follicles or sweat glands [41,42]. Other research also indicated that full-thickness wounds heal not only by re-epithelialization alone [43] because many cellular factors/constituents are involved in the restoration of normal mechanical strength of the epidermis, such as the proliferation and differentiation of keratinocyte stem cells, presence of follicular or sebaceous stem cells, and reformation of an intact basement membrane with associated basal cells. All of these cells are also suggested to contribute to epithelial hyperplasia [44]. It was previously asserted that fatty acids also act as a building block for complex sebum lipids formed by sebaceous glands, and sebum lipids give the skin surface self-disinfecting activity, and these acids are responsible for this property [45]. In agreement with this idea, we reported that the histopathological sections exhibited clear development of sebaceous glands in the treated group and their absence in the control group. As a consequence, sebaceous gland development might contribute to skin immune defense.

## Conclusion

The current work illustrated that *H. tuberosus* powder exerts antibacterial activity along with an effect on promoting wound healing. Thus, it can be used as a conventional topical antibiotic at a low cost from natural sources. In addition, given its content of fatty acids as active ingredients, *H. tuberosus* powder can enhance full-thickness wound contraction. Further studies should investigate the effect of *H. tuberosus* on the gene expression of several cytokines and growth factors during the healing process.

## Authors’ Contributions

NHRA: Designed the study, wrote the manuscript, and participated in conducting the experiment. AGA: Collected the samples, performed the laboratory investigations, processed and analyzed the data. Both authors have read and approved the final manuscript.
